# Design, Synthesis and Adsorption Evaluation of Bio-Based Lignin/Chitosan Beads for Congo Red Removal

**DOI:** 10.3390/ma15062310

**Published:** 2022-03-21

**Authors:** Xiaobing Han, Rong Li, Pengpai Miao, Jie Gao, Guowen Hu, Yuan Zhao, Tao Chen

**Affiliations:** Hubei Key Laboratory of Radiation Chemistry and Functional Materials, School of Nuclear Technology and Chemistry & Biology, Hubei University of Science and Technology, Xianning 437100, China; hanxiaobing@hbust.edu.cn (X.H.); l3281043690@163.com (R.L.); zzu666666@126.com (P.M.); hgwpublic@163.com (G.H.); zhyf308@hbust.edu.cn (Y.Z.)

**Keywords:** adsorbent, design, beads, biomass, Congo red

## Abstract

The morphology and intermolecular interaction are two of the most important factors in the design of highly efficient dye adsorbent in the industry. Millimeter-sized, bead-type, bio-based lignin/chitosan (Lig/CS) adsorbent was designed for the removal of Congo red (CR), based on the electrostatic attraction, π-π stacking, and hydrogen bonding, which were synthesized through the emulsification of the chitosan/lignin mixture followed by chemical cross-linking. The effects of the lignin/chitosan mass ratio, initial pH, temperature, concentration, and contact time on the adsorption were thoroughly investigated. The highest adsorption capacity (173 mg/g) was obtained for the 20 wt% Lig/CS beads, with a removal rate of 86.5%. To investigate the adsorption mechanism and recyclability, an evaluation of the kinetic model and an adsorption/desorption experiment were conducted. The adsorption of CR on Lig/CS beads followed the type 1 pseudo-second-order model, and the removal rate for CR was still above 90% at five cycles.

## 1. Introduction

With the rapid development of chemical processing textile industries, plenty of dyes were poured into the ecosystem, leading to the serious pollution of the water resource [1,2]. According to the charge of the molecules, dyes are classified into different categories, such as cationic dye, anionic dye, and nonionic dye. Among these dyes, Congo red (CR) is a typical anionic diazo dye commonly used in textiles and other commercial products [3]. In addition to its challenge in wastewater treatment, CR also shows carcinogenic properties [4]. Due to its availability, efficiency, low cost, high selectivity, and easy operation in a wide range of conditions, the adsorption could be considered a promising approach among various physico-chemical methods. Numerous novel, powder-type (including the microspheres with nano size) adsorbents were developed for the dye adsorption, especially for the bio-based materials [5,6].

Though these powder-type adsorbents have a relatively high adsorption capacity, benefiting from their large surface area and short diffusion distance, they are not suitable for the industrial application in a practical adsorption column. The powder-type adsorbents can be easily lost or blocked, which will disturb the operation process. The morphology of adsorbents is an essential parameter for the adsorption in industry; millimeter-sized spherical adsorbents are the most favorable for adsorption, according to the literature [7,8], since this kind of adsorbent can be easily handled, separated, and recycled. This is the reason why millimeter-sized, bead-type adsorbents were popularly used in the practical adsorption column [9].

Except for the morphology of adsorbents, the intermolecular interaction between the dye and the adsorbents is another important factor in the application [10]. The commercial dyes are an aromatic compound with a negative or positive charge; some dyes also possess a nitrogen- or oxygen-contained functional group, such as Congo red and Rhodamine B [11]. Based on the structural characteristics of the commercial dyes, numerous adsorbents were developed from the synthetic and natural polymers [12]. In the construction of polymeric adsorbents for dye removal, electrostatic attraction was utilized most popularly due to its ionic characteristic. Among these ionic polymers, the positive chitosan and negative sodium carboxymethyl cellulose/alginate were the most promising candidates [13,14,15,16]. As all of the dyes are aromatic compounds, adsorbents based on π-π stacking were also developed for the removal, including the lignin- and tannin-based adsorbents [17,18]. Another intermolecular interaction involved in the construction of polymeric adsorbents is a hydrogen bond, which can be easily formed between the nitrogen- or oxygen-contained functional group. The combination of the electrostatic attraction, π-π stacking, and hydrogen bonding in the design of adsorbents can improve the adsorption capacity; however, most of the adsorbents were designed based on only one kind of the intermolecular interactions [19,20].

On the other hand, to meet the demand of sustainable development, the renewable, sustainable, economical, and ecofriendly adsorbents from waste biomass sources were developed for the treatment of dye wastewater [21,22]. Among the biomass sources, chitosan, sodium carboxymethyl cellulose, sodium alginate, lignin, and tannin are the most investigated in the bioabsorbent. However, only one kind of intermolecular interaction was utilized in most investigations, which is not beneficial for the improvement of the adsorption capacity.

In recent years, biomaterials have become the focus of absorbent materials, owing to their high removal efficiency, environmental friendliness, and renewability. Among these biomaterials, chitosan is the only one with a positive charge, which is beneficial for the adsorption of anionic dyes [14]. In addition, lignin is the second-most abundant biomaterial, possessing a three-dimensional network structure, which can adsorb dyes through the π-π stacking and hydrogen bonding interaction [17]. Thus, lignin/chitosan-based composites were used for the adsorption of dyes and metal ions in wastewater [11,23,24,25,26,27]. However, most of the obtained lignin/chitosan-based absorbents are powder-type, which is not suitable for the industrial application.

**Figure 1:**
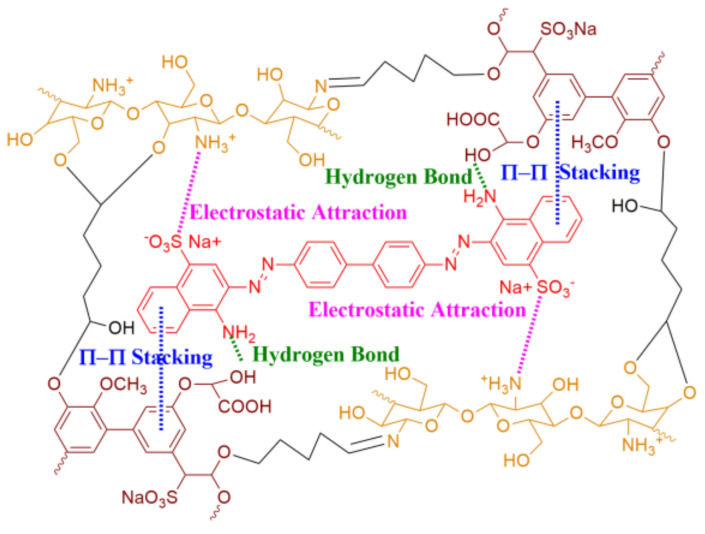
Lignin/chitosan absorbent designed with different intermolecular interactions.

As mentioned above, a combination of the three kinds of intermolecular interactions (electrostatic attraction, π-π stacking, and hydrogen bonding) was designed from a bio-based lignin/chitosan (Lig/CS) absorbent for the removal of Congo red (Figure 1), and a millimeter-sized, bead-type adsorbent was obtained through a method of emulsification followed by chemical cross-linking. The obtained absorbent was used for the removal of Congo red, and the adsorption properties, kinetic model, and recyclability were investigated.

## 2. Materials and Methods

### 2.1. Materials

All reagents were purchased from commercial suppliers of analytical grade reagents and used without further purification. Lignin (Lig, 96%, Mw = 10,000 g/mol), chitosan (CS, deacetylation degree >95%), glacial acetic acid (HAc, 99.5%), glutaraldehyde (50% *v*/*v*), and Congo red (CR, 98%) were purchased from HWRK Chemical Co., Ltd. (Beijing, China). Petroleum ether, liquid paraffin wax, and Tween-80 were supplied by Sinopharm Chemical Reagent Co., Ltd. (Shanghai, China). Distilled water was used throughout the experiments for solution preparation.

### 2.2. Preparation of Bio-Based Lignin/Chitosan (Lig/CS) Beads

**Figure 2:**
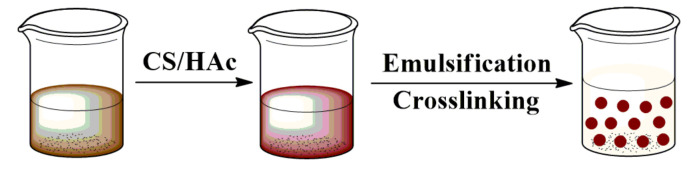
Synthesis procedure of the bio-based Lig/CS beads.

The bio-based lignin/chitosan beads were prepared as follows [13,28] (Figure 2): the lignin was dispersed into 28.5 mL water using a bath sonicator (Kunshan Ultrasound Instrument Co., Ltd., Kunshan, China) for 1 h. Then, 1.5 mL acetic acid and 0.6 g chitosan were added to the lignin dispersion under stirring at 50 °C until complete dissolution (the weight percentage of lignin to chitosan is 0, 10, 20, 40 wt%). Under high-speed stirring at 50 °C, the mixture was then poured into an emulsifier dispersion, which consisted of 1.2 mL Tween-80, 25 mL liquid paraffin wax, and 35 mL petroleum ether. A quarter of an hour later, 1 mL 50% (*v*/*v*) glutaraldehyde was dropped within 30 min for the chemical cross-linking. After reacting for another hour, the Lig/CS beads were obtained with vacuum filtration and washed three times with petroleum ether and water, respectively.

### 2.3. Characterization

The morphologies were observed by a scanning electron microscopy (SEM, VEGA-3, Tescan, Czech Republic). Fourier transform infrared (FTIR) spectra were recorded on an Avatar 360 Nicolet instrument (Thermo Fisher Scientific, Shanghai, China) using KBr pellets, in the wave numbers ranging from 4000 to 400 cm^−1^ with a resolution of 4 cm^−1^. The thermogravimetry (TG) analysis was conducted via a NETZSCH TG 209F3 instrument (NETZSCH Scientific Instruments Trading, Ltd., Shanghai, China) under an N_2_ atmosphere with a heating rate of 10 °C/min^−1^. UV-Vis absorption spectra were conducted with a S 3100 spectrophotometer (Mapada Instruments Co., Ltd., Shanghai, China).

### 2.4. Adsorption Experiments

Adsorption experiments were performed in a 100 mL Erlenmeyer flask; the Lig/CS beads (containing 30 mg adsorbent) were added to 30 mL of the Congo red (CR) solution, and then the solution was agitated under 150 rpm in an incubator shaker. The pH of the solution was adjusted with a 0.1 M NaOH and 0.1 M HCl solution. The concentration of CR in the final solution was determined by using a UV-vis spectroscopy at 497 nm. The adsorption capacity *Q* (mg/g) and removal efficiency *R* (%) was calculated as follows:

(1) $$Q=\frac{(C_{0}-C_{t})V}{W}$$

(2) $$R=\frac{C_{0}-C_{t}}{C_{0}}\times 100$$

where *C*_0_ (mg/L) and *C_t_* (mg/L) are the initial and final concentrations of CR in the solution, *W* (g) is the weight of the Lig/CS beads, and *V* (L) is the volume of the adsorption solution.

## 3. Results

### 3.1. Synthesis and Characterization of Lig/CS Beads

Chitosan beads and lignin beads can be easily formed in the presence of a glutaraldehyde cross-linking agent [29,30]. The synthesis of the Lig/CS beads is schematically presented in Figure 2, according to the design in Figure 1.

#### 3.1.1. Morphology Analysis

**Figure 3:**
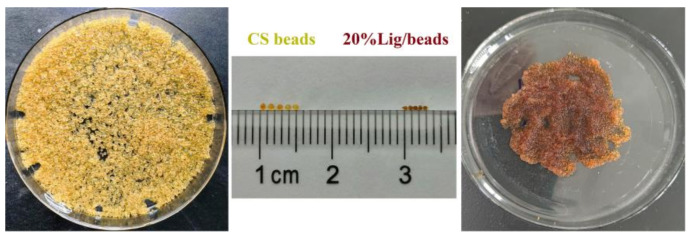
Image of the pure CS beads and 20% Lignin/Chitosan (Lig/CS) beads.

The image of the obtained pure CS beads and 20% Lig/CS beads are shown in Figure 3. It can be clearly observed that the obtained composites are a spherical shape and show a uniform size distribution. The obtained pure CS beads exhibit a light yellow colour with a diameter of about 1 mm while the color changed to yellowish-brown and the diameter decreased to about 0.6 mm with the introduction of 20% lignin. The morphology of the obtained composites were millimeter-sized beads, similar to the commercial macroporous adsorption resin [31], which will benefit from the handling, separating, and recycling in an industrial application [32,33].

**Figure 4:**
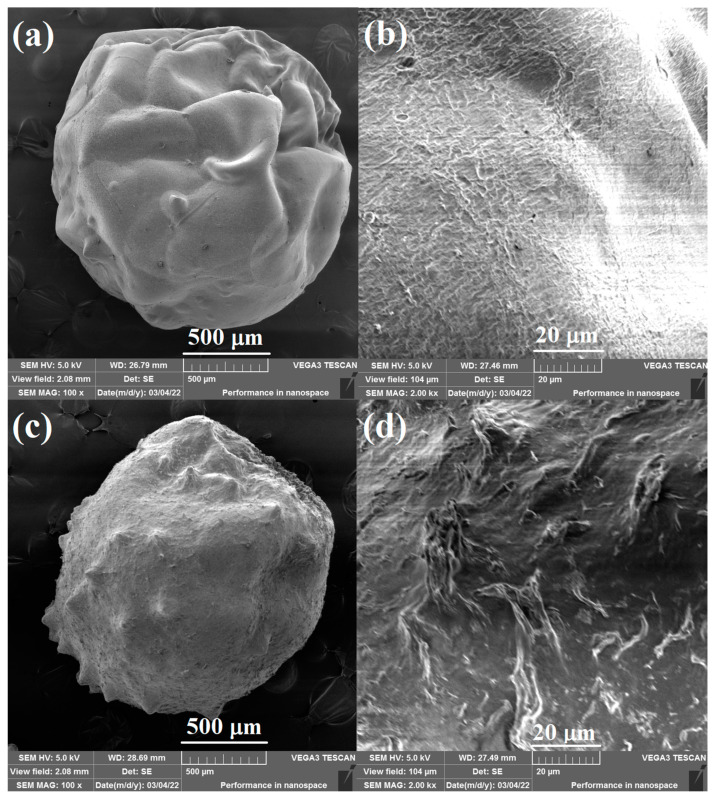
SEM images of the pure CS beads (**a**,**b**) and 20% Lig/CS beads (**c**,**d**) with different resolution.

The surface morphology of the pure CS beads and 20% Lig/CS beads are shown in Figure 4. The obtained adsorbents are spherically shaped, which is different from the powder-type lignin/chitosan composites [11,25]. The pure CS beads show a smooth surface (Figure 4a,b) while the 20% Lig/CS beads present a rough surface with many folds. The introduction of lignin into the chitosan matrix enhances the surface area of the obtained beads, which will benefit from the adsorption of dyes [27].

#### 3.1.2. Composition Analysis

**Figure 5:**
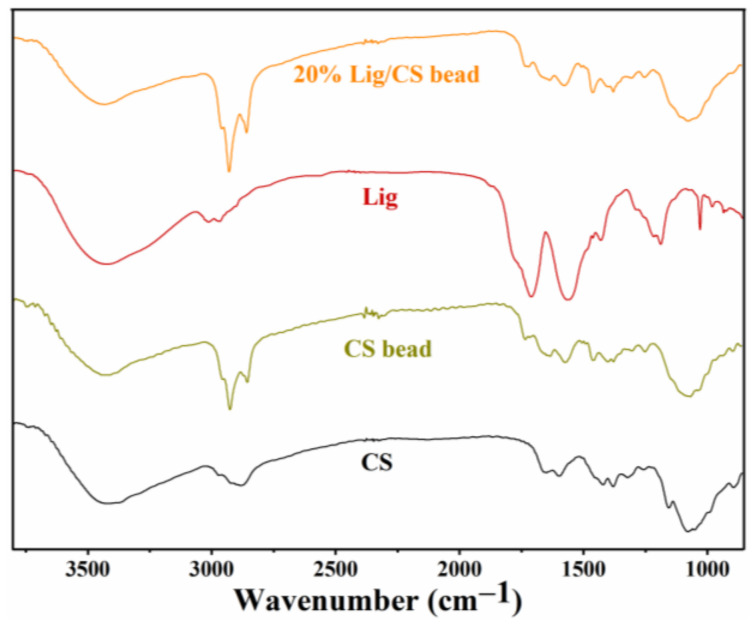
FTIR spectra of the CS, CS beads, Lig, and 20% Lig/CS beads.

The FTIR spectra of CS, CS beads, lignin, and 20% Lig/CS beads are shown in Figure 5. In the spectra of CS, stretching vibrations of the -OH and -NH_2_ groups and intermolecular hydrogen bonds were confirmed with the broad peak from 3300 to 350 cm^−1^. The presence of the stretching vibration (2931 cm^−1^) and bending vibration (1375 cm^−1^) of CH_3_ as well as the C−N stretching vibration (1650 cm^−1^) of amide reveal the existence of an unremoved acetyl group. In addition, the peaks at 1157 and 1092 cm^−1^ can be ascribed to the C−OH bending vibration and C−O−C stretching vibration [29]. Compared to the pure CS, an obvious change was observed in the FTIR spectra of CS beads. The peak referring to the C−OH disappeared; a new peak for imines (C=N) at 1690 cm^−1^ formed, and an increased intensity for methylene (2861 cm^−1^) was observed, which confirms the cross-linking of CS with glutaraldehyde [34]. For the spectra of Lig, the wide band centered at 3450 cm^−1^ is attributed to aliphatic and phenolic -OH. The peaks at 2972 and 2920 cm^−1^ are ascribed to the stretch vibration of methyl and methylene; 1610 and 1461 cm^−1^ is the C−C stretching for the aromatic skeleton, and 1314 and 1161 cm^−1^ is attributed to the vibration of S=O. For the 20% Lig/CS beads, all the peaks of CS, Lig, and the characteristic peak of cross-linking can be observed, demonstrating the successful preparation of Lig/CS beads [29,30].

#### 3.1.3. Thermogravimetric Analysis

**Figure 6:**
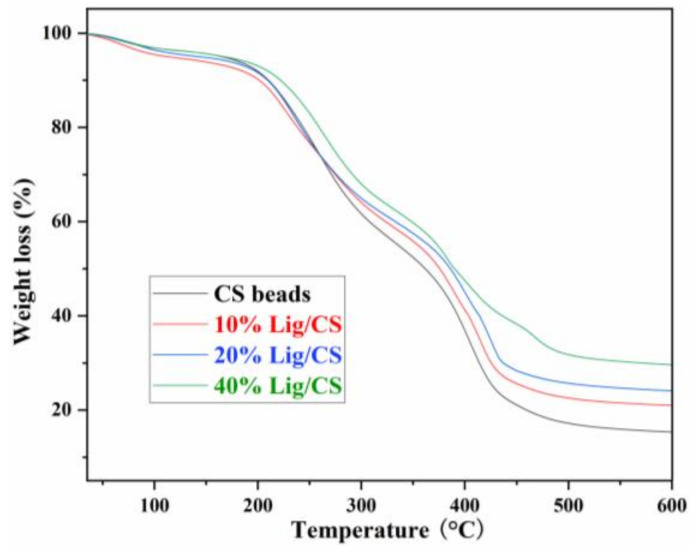
TG spectra of the Lig/CS beads with different lignin content.

Thermalgravimetric analysis (TG) curves can describe the trend of materials degradation with an increase in temperature and reveal the thermal stability of the materials. The TG curves of CS beads with different Lig content are shown in Figure 6. The pure CS beads show obvious degradation from 200 °C, higher than that of raw CS [11], revealing that the chemical cross-linking with glutaraldehyde is a useful approach to enhancing the thermal stability of the CS. The pure CS beads show two main weight loss stages in the ranges of 200–300 °C and 360–430 °C, which is consistent with the results in the literature [13]. With the increase of lignin content in the Lig/CS beads, the amount of residue increased from 17.2% to 21.5%, 25.7%, and 31.7% at 500 °C, respectively, demonstrating that the thermal stability of the obtained Lig/CS beads became higher. This can be ascribed to the aromatic structure in the lignin molecule, which is more stable than the aliphatic chain of CS [11].

### 3.2. Adsorption and Regeneration Behaviour of Lig/CS Beads

Congo red (CR) is a general commercial dye, which possesses a negative charge (sulfonate), aromatic ring (biphenyl and naphthyl), and functional group (amino) [20]. As discussed in the composition analysis (Figure 3), the obtained Lig/CS beads possess easily protonated amino (CS), an aromatic ring (Lig), and a nitrogen (-NH_2_)- and oxygen (-OH)-contained functional group. According to the literature [13,14,15,16,17,18,19,20], the CR can be adsorbed onto Lig/CS beads through the combination of electrostatic attraction, π-π stacking, and hydrogen bonding. The obtained Lig/CS beads were used for the adsorption of CR under different conditions.

#### 3.2.1. Effect of Lignin Content, pH and Temperature

**Figure 7:**
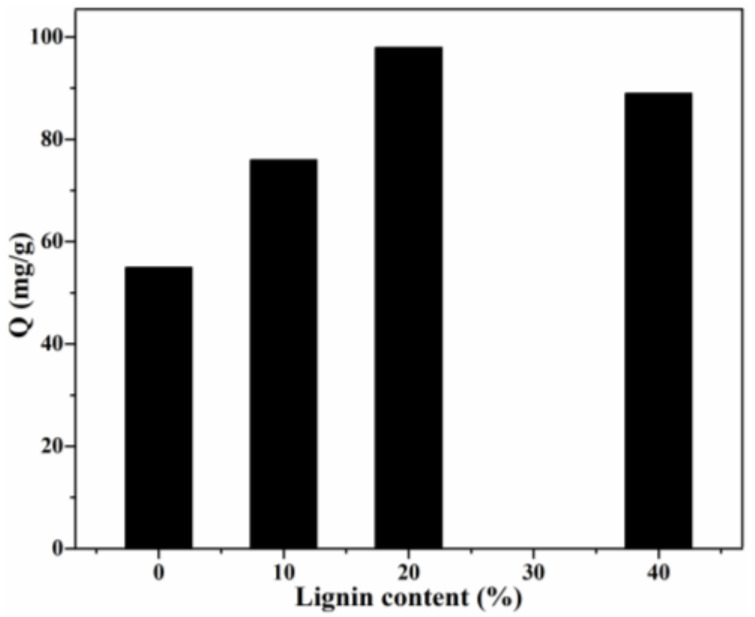
Effect of lignin content on the adsorption capacity. (*C*_0_ = 200 mg/L, pH = 7, T = 25 °C, t = 24 h).

The adsorption capacity of CR by Lig/CS beads with different lignin content is shown in Figure 7. Obviously, with the introduction of aromatic lignin, the adsorption capacity of Lig/CS beads was remarkably enhanced with the increasing lignin content. In particular, the highest adsorption capacity (98 mg/g) was obtained for the Lig/CS beads containing 20% lignin, with a removal rate of 49%. The maximum adsorption capacity of Lig/CS beads is increased by 78% compared with pure CS beads, which can be ascribed to the strong interactions, such as π-π stacking and hydrogen bonding, between the lignin component and the CR molecules [19,20]. With a further increase in lignin content, the adsorption capacity decreased, which may be attributed to the hindered and diffused dye molecule that came from the exceedingly rigid lignin. This is consistent with the results of chitosan/graphene composite spheres for the efficient adsorption of methyl orange [35]. In order to further optimize the adsorption process, the following experiments were conducted in detail by choosing 20% Lig/CS beads as a representative.

**Figure 8:**
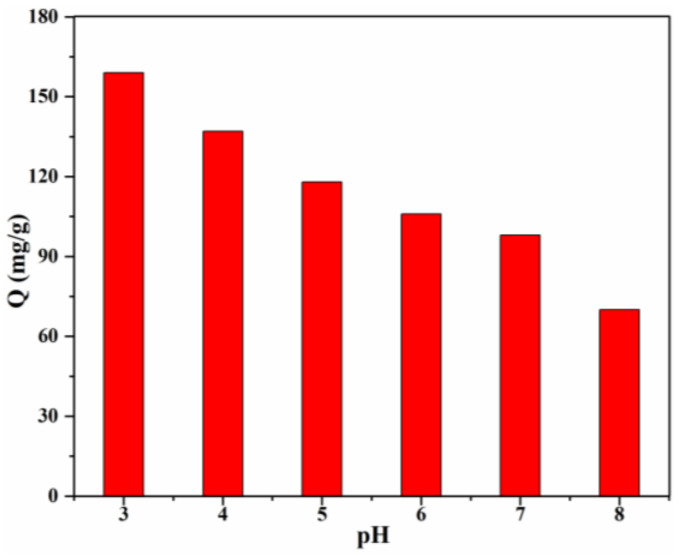
Effect of the initial pH on the adsorption capacity with 20% Lig/CS beads. (*C*_0_ = 200 mg/L, T = 25 °C, t = 24 h).

The solution pH, which influences the adsorption behavior, is one of the most important factors. The effect of pH value of the CR solution on the adsorption capacity was investigated (Figure 8), and the results clearly revealed the pH-dependent adsorption behaviour of 20% Lig/CS beads toward CR, which demonstrated that the electrostatic attraction played an important role in the removal of anionic CR [13]. The highest adsorption capacity (159 mg/g) was obtained for the Lig/CS beads containing 20% lignin at a pH = 3, with a removal rate of 79.5%. This can be ascribed to the positive effect of a high H^+^ concentration; the -NH_2_ and -OH groups on the Lig/CS beads can be easily protonated at a low pH value, which can promote the adsorption of anionic CR through electrostatic attraction. Taking the protection of the environment and equipment into account, adsorption at a very low pH value (<3) was not conducted. With the increase of the pH value, the adsorption capacity of the beads decreased gradually. This can be assigned to the suppression of protonation, which is unfavorable to the removal of CR. When the pH value exceeded 7, the adsorption capacity and removal rate were decreased further. This can be attributed to the suppression of electrostatic attraction and the competitive adsorption of -OH with anionic CR towards Lig/CS beads [35].

**Figure 9:**
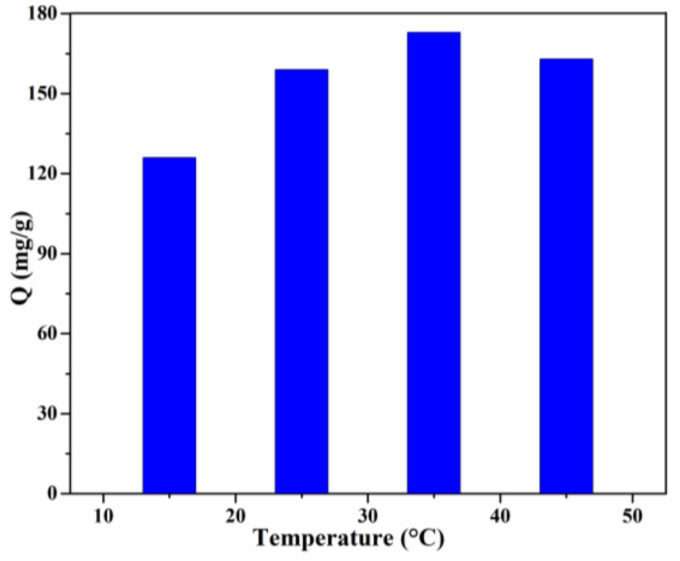
Effect of the temperature on the adsorption capacity with 20% Lig/CS beads. (*C*_0_ = 200 mg/L, pH = 3, t = 24 h).

Temperature is another significant factor which can reveal whether the adsorption is exothermic or endothermic [3]. The effect of the solution temperature on the adsorption capacity is shown in Figure 9. In the temperature range of 15–35 °C, the adsorption capacity increased gradually. The highest adsorption capacity (173 mg/g) was obtained for the Lig/CS beads containing 20% lignin at T = 35 °C, with a removal rate of 86.5%. This result revealed that the adsorption is an endothermic process, which may be attributed to the increase of the mobility of the CR molecules and the number of active sites of Lig/CS beads [36]. With a further increase of the solution temperature, the adsorption capacity decreased, which may be ascribed to the decrease of the intermolecular interaction with the increase of temperature [37].

The thermodynamic parameters, including equilibrium constant *K*^0^, change in the Gibbs free energy (Δ*G*^0^), enthalpy (Δ*H*^0^), and entropy (Δ*S*^0^) and can be calculated by the following Equations (3)–(5) [20,38,39]:

*K*^0^ = *Q_e_*/*C_e_*, (3)

Δ*G*^0^ = −*RT ×* ln *K^0^* (4)

ln *K*^0^ = −Δ*H*^0^/*RT* + Δ*S*^0^*/R* (5)

where *Q_e_* is the adsorption capacity (mg/g), *C_e_* is the equilibrium concentration, *R* is the universal gas constant (8.314 J/mol·K), and *T* is the absolute temperature (K). The Δ*H*^0^ and Δ*S*^0^ are calculated from the slope and intercept of the linear plot of ln *K*^0^ versus 1/*T*.

The calculated results are listed in Table 1. As shown in the table, when the temperature increases from 288 to 308 K, Δ*G*^0^ was decreased from −1.31 to −4.61 kJ/mol, indicating that the high temperature is beneficial for the adsorption, which is consistent with the effect of temperature on the adsorption [38]. In addition, the negative values of Δ*G*^0^ for adsorption indicated the adsorption was a spontaneous physical process [39]. The Δ*H*^0^ and Δ*S*^0^ calculated from the slope and intercept was 8.84 kJ/mol and −0.169 J/mol·K, respectively, and the positive value of Δ*H*^0^ revealed an endothermic nature of adsorption again.

#### 3.2.2. Effect of Concentration and Time

**Figure 10:**
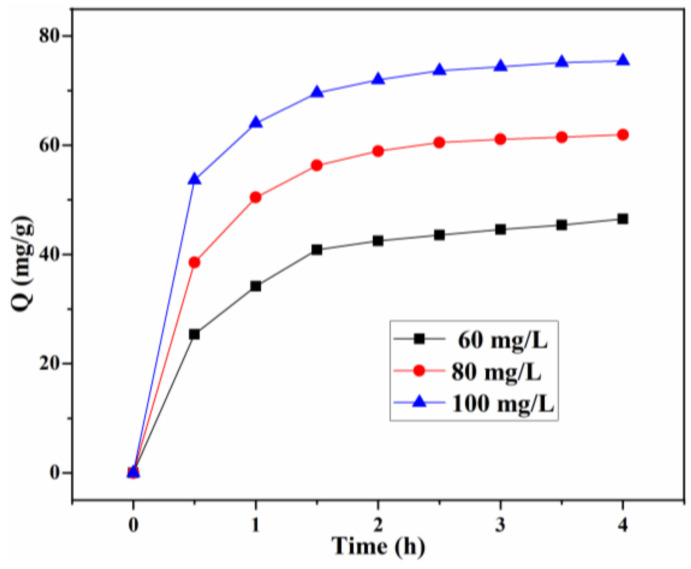
Effect of the initial concentration and contact time on the adsorption capacity Q (mg/g) with 20% Lig/CS beads. (pH = 3, T = 35 °C).

The effect of the initial concentration (60, 80, 100 mg/g) on the adsorption of CR at different times is depicted in Figure 10. The adsorption rates are relatively fast for all investigated concentrations, and the adsorption capacity at equilibrium for different initial concentrations is 46.50, 61.93, and 75.43 mg/g, respectively. Particularly for the adsorption at a low initial concentration (60 mg/L), 1.5 h of the contact time is sufficient for the CR adsorption to reach equilibrium. This can be attributed to a uniform dispersion of bio-based Lig/CS beads in the CR solution, improving the efficient contact with CR molecules and facilitating the mass transfer of CR. When the initial concentration increased to 80 and 100 mg/L, the equilibrium times were about 2 h and 2.5 h, respectively, which revealed that the CR adsorption from the solution is dependent on the initial concentration. After the saturation adsorption on the surface, it takes a relatively long time to diffuse into the internal surface for CR molecules [11,27].

#### 3.2.3. Adsorption Kinetic

Adsorption kinetic is one of the most important characteristics, which can reveal the adsorption efficiency and determine the potential application. In order to investigate the adsorption process and mechanism for CR adsorption onto Ling/CS beads, three kinetic models were used to evaluate the adsorption kinetic process, including the pseudo-first-order model, type 1 pseudo-second-order model, and type 2 pseudo-second-order model (Table 2).

**Figure 11:**
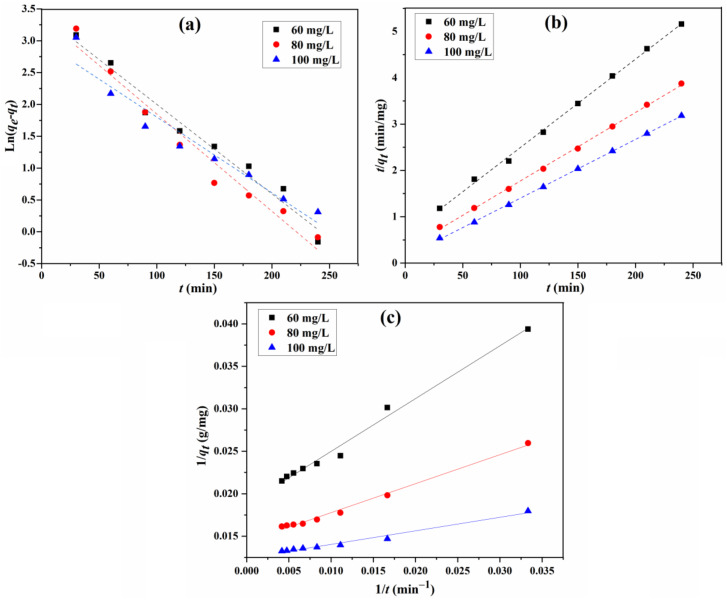
Fitting of pseudo-first-order (**a**), type 1 pseudo-second-order (**b**), and type 2 pseudo-second-order (**c**) kinetic models for the adsorption of CR.

The fitting curves of the adsorption for the three models are displayed in Figure 11, and the parameters, including calculated equilibrium adsorption capacities, rate constants, and correlation coefficients, are calculated from these curves and summarized in Table 3. For the adsorption capacity at the equilibrium for different initial concentrations, the practical determined value is very close to the theoretical calculated one for the type 1 pseudo-second-order model. In addition, the correlation coefficient R^2^ (0.998 to 0.9998) shows that the type 1 pseudo-second-order model fits the experiment data better than that of the other two, suggesting that the type 1 pseudo-second-order mechanism is predominant in the adsorption process, and the chemisorption might have been the rate-limiting step for the adsorption process. In this mechanism, the CR molecule stuck to the adsorbents tended to find sites that maximized the coordination number with the surface [13,40].

#### 3.2.4. Regeneration Behaviour

**Figure 12:**
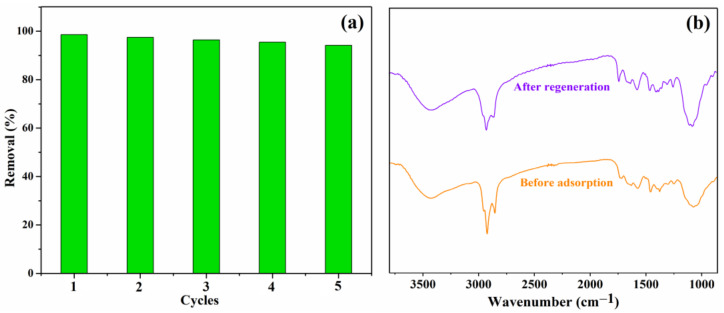
(**a**) Effect of recycling times on the adsorption capacity Q (mg/g) with 20% Lig/CS beads (*C*_0_ = 10 mg/L, pH = 3, T = 35 °C, t = 4 h); (**b**) FTIR spectra of 20% Lig/CS beads before adsorption and after regeneration.

The regeneration is another significant factor in evaluating the properties of the obtained adsorbents; thus, the Lig/CS beads were evaluated by an adsorption/desorption experiment, and the CR-loaded beads were regenerated with a 20% NaOH aqueous solution in an incubator shaker [35]. The adsorption activity of the Lig/CS beads after repeated use was assessed in five successive CR adsorptions, under the identical experimental conditions. As shown in Figure 12a, the removal rate decreased slightly; there was over a 90% removal for the CR observed after five cycles. The results demonstrate that no obvious decrease was observed for the CR removal after regeneration, indicating that the absorbents have a good reusability [11,13]. As shown in Figure 12b, no obvious difference was observed in the FTIR spectra of 20% Lig/CS beads before adsorption and after regeneration, which demonstrated that the absorbent shows a good stability.

#### 3.2.5. Comparative Analysis of CR Adsorption

To determine the efficiency of the obtained Lig/CS adsorbent for the removal of CR, the maximum adsorption capacities, removal, regeneration, and morphology of these adsorbents were compared (Table 4) [11,20,39,41,42,43]. Although some reported adsorbents show relatively high adsorption capacities at high initial concentrations, the removal rate is relatively low, and some adsorbents possess a lower removal rate after regeneration, making regeneration even harder. In addition, most of the obtained adsorbents are not bead-type, which is difficult to apply in commercial adsorption columns.

## 4. Conclusions

In summary, different from conventional powder-type, lignin/chitosan-based absorbents, the millimeter-sized, bead-type lignin/chitosan (Lig/CS) adsorbent was successfully synthesized for the removal of Congo red, which will benefit the industrial application. The introduction of lignin in chitosan can not only enhance the stability of the beads but also can improve the adsorption capacity through the strong π-π stacking interaction between the lignin component and the CR molecules. The optimum content of lignin is 20%; a higher content of lignin will hinder the diffusion of CR molecules. A lower pH value can increase the adsorption capacity, which can be ascribed to the enhanced electrostatic attraction. The experiments and corresponding calculation revealed that the adsorption of CR on Lig/CS beads followed the type 1 pseudo-second-order model. The removal rate for CR is still above 90% after five cycles, and no obvious structural difference was observed for the regenerated adsorbents. Therefore, the bio-based lignin/chitosan beads have great practical potential as an efficient adsorbent for the removal of anionic dyes in industry. Future work will focus on the selectivity, continuous column adsorption, and scale-up of this kind of bio-based bead adsorbent.

## Figures and Tables

**Table 1:** Thermodynamic parameters for the adsorption of Congo red (CR) on 20% Lig/CS beads.

Temperature (K)	ln *K*^0^	Δ*G*^0^ (kJ/mol)	Δ*H*^0^ (kJ/mol)	Δ*S*^0^ (kJ/mol·K)
288	0.53	−1.31	8.84	−0.169
298	1.36	−3.37	-	-
308	1.86	−4.61	-	-

**Table 2:** The kinetic models used to evaluate the adsorption process [11].

Kinetics Models	Pseudo-First-Order	Type 1 Pseudo-Second-Order	Type 2 Pseudo-Second-Order
Equations	$\ln(q_{e}-q_{t})=\ln q_{e}-k_{f}t$	$q=\frac{q_{\max}K_{L}C_{e}}{1+K_{L}C_{e}}$	$\frac{1}{qt}=\frac{1}{qe}+(\frac{1}{k3{qe}^{2}})(\frac{1}{t})$
Rate constant *k*	−slope	slope^2^/intercept	intercept^2^/slope
*q*_e,cal_	*e* ^intercept^	1/slope	1/intercept

**Table 3:** Kinetic parameters of three models for Congo red adsorption.

Adsorption Isotherm Models	Coefficients	60 mg/L	80 mg/L	100 mg/L
Pseudo-first-order	*q*_e,cal_ (mg/g)	34.9878	34.7092	23.8645
	*k*_1_ (L/g)	0.0141	0.0152	0.0118
	*R* ^2^	0.9666	0.9622	0.9298
Type 1 pseudo-second-order	*q*_e,cal_ (mg/g)	52.5486	69.9301	78.8643
	*k*_2_ (×10^−3^) (g/mg min)	0.6161	0.7519	1.206
	*R* ^2^	0.9982	0.9991	0.9998
Type 2 pseudo-second-order	*q*_e,cal_ (mg/g)	53.2765	69.6378	80.3858
	*K*_3_ (g/mg min)	20.5946	8.1166	2.0451
	*R* ^2^	0.9874	0.9917	0.9783

**Table 4:** Comparison of the CR removal performance with other adsorbents.

Adsorbent	*C*_0_ (mg/L)	*Q_max_* (mg/g)	Removal (%)	Regeneration	Morphology	Ref.
Lignin/Chitosan	200	173	86.5	>90% 5 cycles	millimeter-sized bead	This work
Chitosan-Ignosulfonate	1000	450	45.0	ND	powder	[11]
GO/Chitosan	50	125	84.6	ND	pellets	[20]
GO/Cellulose/PNIPAAm	800	695	43.4	84.9	hydrogel	[38]
PPD-GO	800	893	27.9	ND	powder	[41]
Modified Chitosan	696	63	96.6	100% 3 cycles	hydrogel	[42]
Cellulose-chitosan	4000	1170	58.5	hard	foams	[43]

## Data Availability

All the data is available within the manuscript.

## References

[1] Sala E., Mayorga J., Bradley D., Cabral R.B., Atwood T.B., Auber A., Cheung W., Costello C., Ferretti F., Friedlander M.A. (2021). Protecting the Global Ocean for Biodiversity, Food and Climate. Nature.

[2] Sullivan S.M.P., Rains M.C., Rodemald A.D., Buzbee W.W., Rosemond A.D. (2020). Distorting Science, Putting Water at Risk. Science.

[3] Li Y., Meas A., Shan S.D., Yang R.Q., Gai X.K. (2016). Production and Optimization of Bamboo Hydrochars for Adsorption of Congo red and 2-Naphthol. Bioresour. Technol..

[4] Mall I.D., Srivastava V.C., Agarwal N.K., Mishra I.M. (2005). Removal of Congo Red from Aqueous Solution by Bagasse Fly Ash and Activated Carbon: Kinetic study and equilibrium isotherm analyses. Chemosphere.

[5] Srivastava V., Zare E.N., Makvandi P., Zheng X.-Q., Iftekhat S., Wu A., Padil T.V.V., Mokhtari B., Varma S.R., Tay R.F. (2020). Cytotoxic Aquatic Pollutants and Their Removal by Nanocomposite-based Sorbents. Chemosphere.

[6] Zhou Y.B., Lu J., Zhou Y., Liu Y.D. (2019). Recent Advances for Dyes Removal Using Novel Adsorbents: A Review. Environ. Pollut..

[7] Kim J., Lee H., Vo H.T., Lee G., Kim N., Jang S., Joo J.B. (2020). Bead-Shaped Mesoporous Alumina Adsorbents for Adsorption of Ammonia. Materials.

[8] Wang Z.L. (1999). Effect of Adsorbent Shape on Adsorption Dynamics in A Batch Adsorber. Chem. Eng. Sci..

[9] Hassan M.M., Carr C.M. (2018). A Critical Review on Recent Advancements of the Removal of Reactive Dyes from Dyehouse Effluent by Ion-exchange Adsorbents. Chemosphere.

[10] Villarreal I.A., Arriagada D.C., Mayorga C.K., Urbina K.P., Valencia R.M., Gonzalez J. (2020). Importance of the Interaction Adsorbent-adsorbate in the Dyes Adsorption Process and DFT Modeling. J. Mol. Struct..

[11] Gu F., Geng J., Li M.L., Chang J.M., Cui Y. (2019). Synthesis of Chitosan-Ignosulfonate Composite as an Adsorbent for Dyes and Metal Ions Removal from Wastewater. ACS Omega.

[12] Han X.B., Gao J., Chen T., Zhao Y. (2020). Interfacial Interaction and Steric Repulsion in Polymer-assisted Liquid Exfoliation to Produce High-quality Graphene. Chem. Pap..

[13] Chen T., Zhao Y., Sang Y.N., Tang M., Hu G.W., Han X.B., Gao J., Ma R. (2021). Facile synthesis of magnetic CS-g-SPSS microspheres via electron beam radiation for efficient removal of methylene blue. J. Saudi Chem. Soc..

[14] Suginta W., Khunkaewla P., Schulte A. (2013). Electrochemical Biosensor Applications of Polysaccharides Chitin and Chitosan. Chem. Rev..

[15] Gericke M., Trygg J., Fardim P. (2013). Functional Cellulose Beads: Preparation, Characterization, and Applications. Chem. Rev..

[16] Ahmad A., Mubarak N.M., Jannat F.T., Ashfaq T., Santulli C., Rizwan M., Najda A., Bin-Jumah M., Abdel-Daim M.M., Hussain S. (2021). A Critical Review on the Synthesis of Natural Sodium Alginate Based Composite Materials: An Innovative Biological Polymer for Biomedical Delivery Applications. Processes.

[17] Sun Z.H., Fridrich B., Santi A., Elangovan S., Barta K. (2018). Bright Side of Lignin Depolymerization: Toward New Platform Chemicals. Chem. Rev..

[18] Bacelo H.A.M., Santos S.C.R., Botelho C.M.S. (2016). Tannin-based Biosorbents for Environmental Applications—A Review. Chem. Eng. Sci..

[19] Bai H.J., Chen J.H., Zhou X.Y., Hu C.Z. (2020). Single and Binary Adsorption of Dyes from Aqueous Solutions Using Functionalized Microcrystalline Cellulose from Cotton Fiber. Korean J. Chem. Eng..

[20] Du Q.J., Li Y.H., Li J.B., Zhang Z., Qiao B., Sui K., Wang D., Wang C., Li H., Xia Y. (2019). Preparation of Graphene Oxide/Chitosan Pellets and Their Adsorption Properties for Congo Red. Int. J. Nanosci..

[21] Li W., Mu B.N., Yang Y.Q. (2019). Feasibility of Industrial-scale Treatment of Dye Wastewater via Bioadsorption Technology. Bioresour. Technol..

[22] Tamjidi S., Ameri A. (2022). A Review of the Application of Sea Material Shells as Low Cost and Effective Bio-adsorbent for Removal of Heavy Metals from Wastewater. Environ. Sci. Pollut. R.

[23] Wawrzkiewica M., Bartczak P., Jesionowski T. (2017). Enhanced Removal of Hazardous Dye form Aqueous Solutions and Real Textile Wastewater Using Bifunctional Chitin/lignin Biosorbent. Int. J. Biol. Macromol..

[24] Bartczak P., Klapiszewski L., Wysokowski M., Majchrzak I., Czernika W., Piasecki A., Ehrlich H., Jesionowski T. (2017). Treatment of Model Solutions and Wastewater Containing Selected Hazardous Metal Ions Using A Chitin/lignin Hybrid Material as An Effective Sorbent. J. Environ. Manag..

[25] Wysokowski M., Klapiszewski L., Moszynski D., Bartczak P., Szatkowski T., Majchrzak I., Stefanska K.S., Bazhenov V., Jesionowski T. (2014). Modification of Chitin with Kraft Lignin and Development of New Biosorbents for Removal of Cadmium(II) and Nickel(II) Ions. Mar. Drugs.

[26] Zdarta J., Klapiszewski L., Wysokowski M., Norman M., Radzimska A.K., Moszynski D., Ehrlich H., Maciejewski H., Stelling A.L., Jesionowski T. (2015). Chitin-Lignin Material as a Novel Matrix for Enzyme Immobilization. Mar. Drugs.

[27] Duan Y.Q., Freyburger A., Kunz W., Zollfrank C. (2018). Lignin/Chitin Films and Their Adsorption Characteristics for Heavy Metal Ions. ACS Sustain. Chem. Eng..

[28] Zhou Z.K., Lin S.Q., Yue T.L., Lee T.C. (2014). Adsorption of Food Dyes from Aqueous Solution by Glutaraldehyde Cross-linked Magnetic Chitosan Nanoparticles. J. Food Eng..

[29] Nagireddi S., Katiyar V., Uppaluri R. (2017). Pd(II) Adsorption Characteristics of Glutaraldehyde Cross-linked Chitosan Copolymer Resin. Int. J. Biol. Macromol..

[30] Yang W.J., Qi G.C., Kenny J.M., Puglia D., Ma P.M. (2020). Effect of Cellulose Nanocrystals and Lignin Nanoparticles on Mechanical, Antioxidant and Water Vapour Barrier Properties of Glutaraldehyde Crosslinked PVA Films. Polymers.

[31] Zhang Q.X., Zhang Z.P., Li A.M., Pan B.C., Zhang X.L. (2018). Advance in Ion Exchange and Adsorption Resins in China. Acta Polym. Sin..

[32] Ma M.S., Liu Z., Hui L.F., Shang Z., Yuan S.Y., Dai L., Liu P.T., Liu X.L., Ni Y.H. (2019). Lignin-containing Cellulose Nanocrystals/Sodium Alginate Beads as Highly Effective Adsorbents for Cationic Organic Dyes. Int. J. Biol. Macromol..

[33] Wu M., Chen W.J., Mao Q.H., Bai Y.S., Ma H.Z. (2019). Facile Synthesis of Chitosan/gelatin Filled with Graphene Bead Adsorbent for Orange II Removal. Chem. Eng. Res. Des..

[34] Bui T.H., Lee W., Jeon S.B., Kim K.W., Lee Y. (2020). Enhanced Gold(III) Adsorption Using Glutaraldehyde-crosslinked Chitosan Beads: Effect of Crosslinking Degree on Adsorption Selectivity, Capacity, and Mechanism. Sep. Purif. Technol..

[35] Zhang C.L., Chen Z.Z., Guo W., Zhu C.W., Zou Y.J. (2018). Simple Fabrication of Chitosan/Graphene Nanoplates Composite Spheres for Efficient Adsorption of Acid Dyes from Aqueous Solution. Int. J. Biol. Macromol..

[36] Senthilkumaar S., Kalaamani P., Subburaam C.V. (2006). Liquid Phase Adsorption of Crystal Violet onto Activated Carbons derived from Male Flowers of Coconut Tree. J. Hazard. Mater..

[37] Ofomaja A.E., Ho Y.S. (2007). Equilibrium Sorption of Anionic Dye from Aqueous Solution by Palm Kernel Fibre as Sorbent. Dye Pigment..

[38] Zhang L.J., Zhao D.Q., Lu Y., Chen J.H., Li H.T., Xie J.H., Xu Y., Yuan H.K., Liu X.J., Zhu X.Y. (2021). A Graphene Oxide Modified Cellulose Nanocrystal/PNIPAAm IPN Hydrogel for the Adsorption of Congo Red and Methylene Blue. New J. Chem..

[39] Zhu H.Y., Fu Y.Q., Jiang R., Jiang J.H., Xiao L., Zeng G.M., Zhao S.L., Wang Y. (2011). Adsorption Removal of Congo Red onto Magnetic Cellulose/Fe_3_O_4_/Activated Carbon Composite: Equilibrium, Kinetic and Thermodynamic Studies. Chem. Eng. J..

[40] Chen T., Yan C.J., Wang Y., Tang C., Zhou S., Zhao Y., Ma R., Duan P. (2015). Synthesis of Activated Carbon-based Amino Phosphonic Acid Chelating Resin and Its Adsorption Properties for Ce(III) Removal. Environ. Technol..

[41] Wu Z.L., Liu F., Li C.K., Chen X.Q., Yu J.G. (2016). A Sandwich-structured Graphene-based Composite: Preparation, Characterization, and its Adsorption Behaviors for Congo Red. Colloid Surf. A Physicochem. Eng. Asp..

[42] Mohamed N.A., Harby N.F., Almaeshed M.S. (2020). Enhancement of Adsorption of Congo Red Dye onto Novel Antimicrobial Trimellitic Anhydride Isothiocyanate-cross-linked Chitosan Hydrogels. Polym. Bull..

[43] Kim U.J., Kim D., You J., Choi J.W., Kimura S., Wada M. (2018). Preparation of Cellulose-chitosan Foams Using an Aqueous Lithium Bromide Solution and Their Adsorption Ability for Congo Red. Cellulose.

